# Growth and growth curve analysis in Dorper × Tumele crossbred sheep under a smallholder management system

**DOI:** 10.1093/tas/txad034

**Published:** 2023-04-17

**Authors:** Belay Deribe, Zeleke Tesema, Mesfin Lakew, Asres Zegeye, Alemu Kefale, Mekonnen Shibesh, Liuel Yizengaw, Negus Belayneh

**Affiliations:** Sirinka Agricultural Research Center, Woldia, Ethiopia; Debre Birhan Agricultural Research Center, Debre Birhan, Ethiopia; Amhara Agricultural Research Institute, Bahir Dar, Ethiopia; Sirinka Agricultural Research Center, Woldia, Ethiopia; Sirinka Agricultural Research Center, Woldia, Ethiopia; Sirinka Agricultural Research Center, Woldia, Ethiopia; Sirinka Agricultural Research Center, Woldia, Ethiopia; Andasa Livestock Research Center, Bahir Dar, Ethiopia

**Keywords:** growth curve, Kleiber ratio, live weight, nonlinear models, weight gain

## Abstract

This study aimed to evaluate the growth performance and Kleiber ratio (KR) and to determine the growth curve of Dorper × Tumele sheep under a smallholder management system. Growth and efficiency-related traits were analyzed by using the general linear model (GLM) procedure of SAS. Gompertz, Logistics, Brody, Monomolecular, and Negative exponential models were used to determine the growth curve, and growth curve parameters were estimated via the nonlinear regression model (NLIN) procedure of SAS. The overall least-squares means of the birth weight, weaning weight, 6-month weight, and yearling weight were 3.29, 13.7, 17.3, and 23.4 kg, respectively. Dorper × Tumele lambs grew faster during the preweaning period (115.3 ± 1.19 g day^−1^) than during the postweaning periods (44.1 ± 1.26 g day^−1^ to 33.5 ± 1.13 g day^−1^). Likewise, a higher KR was observed during the pre-weaning age (16.1 ± 0.08 g/day/kg^0.75^) than during postweaning periods (5.08 ± 0.13 g/day/kg^0.75^ to 3.10 ± 0.09 g/day/kg^0.75^). Brody, a model without an inflection point was the best-fitted growth function for Dorper × Tumele sheep under a smallholder management system. The highest and lowest asymptotic weight was observed for Brody (23.8 ± 0.22 kg) and Logistics (20.7 ± 0.11 kg) models, respectively. The maturation rate ranged between 0.21 (Brody) and 0.66 (Logistics). Based on the Brody model, the correlation between asymptotic weight and maturity rate was −0.92. The growth parameter estimate in this study indicates that Dorper × indigenous sheep had a better speed to achieve mature weight and the early mature crossbred sheep are less likely to exhibit high adult weight. The rapid growth of crossbred sheep during the early period can provide more profit to the farmer by reducing the cost of sheep production inputs. Therefore, crossing Tumele with Dorper sheep and integrating with improved management would be suggested to improve productivity and profit from sheep production.

## INTRODUCTION

Ethiopian farming practices heavily rely on the production of sheep. The sheep population in Ethiopia is estimated to be about 31.30 million and out of this 99.81% of the population is indigenous breeds (CSA, 2018). Resource-poor farmers can afford to keep sheep on smaller land and improve their economic and nutritional status. Sheep are a significant source of revenue, food (meat and milk) for rural households, and raw materials for industry (skins and wool). Besides, sheep contribute socioeconomic and cultural functions, risk-mitigating during crop failures, increase property security, and serve as a form of investment (Tibbo, 2006). Sheep account for 34% of exports of live animals, making them another key source of foreign exchange (Gizaw et al., 2013).

Indigenous breeds are not seasonal breeders, produce under harsh environments, are heat tolerant, resistant/tolerant to disease, and ability to use poor-quality feed. Despite the large population and better adaptability, the importance of indigenous sheep both to the livelihood of resource-poor farmers and to the national economy, the current level of productivity in the smallholder production systems is low (Getachew and Mohamadou, 2014). Consequently, to improve the productivity of indigenous sheep breeds through crossbreeding, Dorper sheep were imported from South Africa in 2007. This breed is a crossbreeding result of Black-headed Persian and Dorset Horn (Cloete et al., 2000). This breed became well-known around the world for its great body conformation, rapid growth, and high-caliber carcass. They can attain a promising weight at an early age (3 to 4 months) and reach to market as compared to indigenous breeds. These fast-growing breeds can play a great contribution to the food sufficiency of smallholder farmers. Because of this, crossbreeding Dorper with Ethiopian indigenous sheep has been conducted and the resulted in crossbreds were disseminated to farmers since 2012 to improve meat production through crossbreeding.

Evaluation of the growth performance and Kleiber ratio (KR) of crossbreds under extensive management is crucial since it is an indicator of their real potential and adaptability. Besides, knowledge of the growth curve is quite important for efficient animal management and a sound basis for developing a breeding strategy to modify the trajectory of growth (Abegaz et al., 2010; Ayied et al., 2011; Tariq et al., 2011; Saghi et al., 2012). However, there is limited information on the growth performance and KR (Mohammadi et al., 2010; Mandal et al., 2015; Tesema et al., 2022) and many studies have been devoted to model the growth curve and growth parameter estimates growth curve of different sheep breeds (Kopuzlu et al., 2014; Lupi et al., 2016; Balan et al., 2017; Hojjati and Hossein-Zadeh, 2018; Iqbal et al., 2019; Mohammadi et al., 2019). The growth and growth curve of animals, however, are influenced by both genetic and nongenetic factors (Kopuzlu et al., 2014; Do and Miar, 2019) and vary depending on the breed. To the best of our knowledge, the nonlinear growth models have not yet been implemented for the estimation of growth curve parameters in Dorper × Tumele sheep. Therefore, this study aimed to evaluate the growth performance and KR and also to determine the growth curve of Dorper × Tumele sheep under a smallholder management system.

## MATERIALS AND METHODS

### Description of the Study Area

The study was conducted in Raya Kobo district, which is located in northern Ethiopia at 570 km from the capital Addis Abeba. The site is located at an altitude of 1,470 m.a.s.l and the rainfall pattern is bimodal, with the two-rainfall season, *belg* (February/March to April) and *kiremt* (July to September). The mean annual rainfall amount is about 630 mm and the temperature varies from 19 to 33 °C with a mean annual temperature of 23.1 °C.

### Community-Based Crossbreeding Modality

The community-based crossbreeding program was initiated in the year 2012 to improve the productivity of sheep through crossbreeding. Volunteer farmers who have at least five breeding female sheep were selected. The training was given to the farmers about the management, utilization of ram, and the importance of the cross-breeding program. Dorper × Tumele rams with 50% blood level were disseminated to the participant farmers based on the number of ewes, that is, one ram for 10 to 15 ewe ratio. Farmers that had <10 ewes were made a group based on their agreement and proximity. All ewes were ear-tagged for identification purposes and lambs born were also given ear tags 15 days to 1-month age. The recruitment of enumerators for data collection was done and training was given on how to collect the necessary data. The researcher has monitored the village at least one time per month. The ram rotation was done in a 1-year interval to avoid inbreeding.

### Data Collation and Studied Traits

The data were collected from the year 2012 to 2018. The growth trait includes birth weight (BWT), weaning weight (WWT), six-month weight (SMWT), yearling weight (YWT), average daily gain from birth to 3 months (ADG1), 3 months to 6 months (ADG2), 6 to 12 months (ADG3), and the corresponding KR at respective stages of growth. The KR was calculated from birth to 3 months (KR1 = ADG1/WWT^0.75^), 3 to 6 months (KR2 = ADG2/SMWT^0.75^), and 6 to 12 months of age (KR3 = ADG3/YWT^0.75^). The number of records for BWT, WWT, SMWT, and YWT were 407, 362, 305, and 125, respectively. Thus, a total of 1,199 weight-age records were used to determine the growth curve of sheep under a smallholder management system.

### Data Analysis

Least squares means and their corresponding standard errors were obtained for growth trait and KRs using the PROC GLM procedure of SAS (2002) with the model that includes sex, blood level, parity of the ewe, the season of lambing, and year of lambing as fixed effects. The litter size was not considered a fixed factor as twin-born lambs were small in number. The interactions among fixed effects were nonsignificant (*P* > 0.05). The mean separation was done using the Tukey-Kramer test.

The following model equation was used for statistical analysis:


$$\begin{array}{l} Y_{ijklmn}=\mu +S_{i}+B_{j}+P_{k}+M_{l}+Y_{m}+e_{ijklmn} \end{array}$$

where *Y*_*iljklmn*_ is the the live weights, weight gain, and KRs of the *n*th lamb. μ is the overall mean. *S*_*i*_ is the effect of the *i*th sex (female, male). *B*_*j*_ is the effect of the *j*th blood level (Dorper gene inheritances) (25%, 37.5%). *P*_*k*_ is the effect of the *k*th parity of ewe (1, 2, 3, 4, ≥5). *M*_l_ is the effect of the *l*th lambing season (dry, short rain, main rain). *Y*_*m*_ is the effect of the *m*th lambing year (2012 to 2018). *e*_*ijklmn*_ is the residual error.

Nonlinear growth curve model parameters were estimated by Levenberg-Marquardt’s iterative algorithm by using the NLIN procedure of SAS (2002). Five nonlinear functions such as Gompertz (Laird, 1965), Logistics (Nelder, 1961), Brody (Brody, 1945), Monomolecular (Draper and Smith, 1981), and Negative exponential (Brown et al., 1976) were tested for the collected data. The models, their corresponding formulas, and variables are explained as follows:


$$\begin{array}{l} Gompertz:\;W\left(t\right)=A{e^{-}}^{Be-Kt}+\;\varepsilon \; \end{array}$$

$$\begin{array}{l} Logistics:\;W\left(t\right)=A/(1+B{e^{-}}^{Kt})+\;\varepsilon \; \end{array}$$

$$\begin{array}{l} Brody:\;W\left(t\right)=A(1-B{e^{-}}^{Kt})+\;\varepsilon \; \end{array}$$

$$\begin{array}{l} Monomolecular:\;W\left(t\right)=A/(1+e^{B-Kt})+\;\varepsilon \; \end{array}$$

$$\begin{array}{l} Negative\;exponential:W\left(t\right)=A(1-{e^{-}}^{Kt})+\;\varepsilon \; \end{array}$$

where *W*(*t*), live weight at age *t* (month); *A* is asymptotic weight or mature weight; and *B* is an integration constant related to the initial animal weight. The value of *b* is defined by the initial values for *W* and *t*; *K* is the maturation rate, which is interpreted as weight change in relation to mature weight to indicate how fast the animal approaches adult weight.

Four goodness-of-fit criteria were used in this study which were Bayesian information criterion (BIC), Akaike information criterion (AIC), coefficient of determination (*R*^2^), and Root of Mean Square Error (RMSE) and the equation is provided as follows:


$$\begin{array}{l} BIC=NLn\left(\frac{SSE}{n}\right)+pLn\left(n\right),\;AIC=NLn\left(\frac{SSE}{N}\right)+2p \end{array}$$

$$\begin{array}{l} R^{2}=1-\left(\frac{SSE}{SST}\right),\;RMSE=\sqrt{\frac{SSE}{n-P-1}} \end{array}$$

where BIC is the Bayesian information criterion; AIC is the Akaike’s information criterion; *R*^2^ is the coefficient of determination; RMSE is the root mean square error; SSE is the sum of square error; SST is the total sum of the square; *N* is the number of observations (data points); *p* is the number of parameters.

## RESULTS

### Preweaning Growth Performance

The live weight at a specific age for Dorper × Tumele sheep under smallholder management is presented in Table 1. The overall mean value of the BWT, WWT, and preweaning gain were observed to be 3.29 kg, 13.7 kg, and 115.3 ± 1.19 g day^−1^, respectively. The influence of blood level except for BWT was found to be nonsignificant (*P* > 0.05). Lambs with 37.5% exotic blood level had higher BWT than lambs with 25% exotic blood level. The sex of lambs was not an important source of variation for all preweaning growth traits considered in this study. However, the parity of ewes exerted a significant influence on the preweaning growth rate of lambs. Lambs born from third parity ewes grew faster than ≥5 parity ewes. However, sex, blood level, and season did not affect the preweaning growth rate of Dorper crossbred lambs. Lambs born during the main rainy season had higher BWT as compared to lambs born in the dry and short rainy seasons. However, the influence of season on other growth traits was no-significant. Year of birth had a considerable effect on the preweaning live weight and weight gain of crossbred lambs in all subsequent ages. Lambs born in 2014, 2016, and 2018 had higher BWT and WWT than in the other years. The daily weight gain of lambs born in the year 2012 and 2014 was higher than lambs born in the other years.

**Table 1.** Preweaning growth performance of Dorper crosses under extensive management

Source of variation	BWT (kg)		WWT (kg)		ADG1 (g day−^1^)	
	*N*	(LSM ± SE)	*N*	(LSM ± SE)	*N*	(LSM ± SE)
Over all	407	3.29 ± 0.02	362	13.7 ± 0.10	362	115.3 ± 1.19
CV(%)	—	14.3	—	13.5	—	18.9
Blood level		***		ns		ns
25%	349	3.25 ± 0.02	309	13.8 ± 0.11	309	116.2 ± 1.28
37.5%	58	3.51 ± 0.07	53	13.4 ± 0.27	53	110.5 ± 3.25
Parity		ns		ns		*
1	84	3.37 ± 0.06	76	13.7 ± 0.28	76	115.1 ± 3.30^ab^
2	123	3.31 ± 0.05	113	13.8 ± 0.16	113	115.6 ± 1.68^ab^
3	114	3.20 ± 0.04	101	13.9 ± 0.19	101	118.9 ± 2.21^a^
4	53	3.28 ± 0.06	46	13.5 ± 0.36	46	111.2 ± 3.50^ab^
≥5	33	3.35 ± 0.08	26	13.1 ± 0.35	26	108.8 ± 4.03^b^
Season		*		ns		ns
Dry	195	3.22 ± 0.03^b^	170	13.7 ± 0.16	170	116.2 ± 1.83
Main rain	78	3.44 ± 0.05^a^	69	14.1 ± 0.18	69	118.6 ± 2.17
Short rain	134	3.31 ± 0.04^b^	123	13.5 ± 0.20	123	112.4 ± 2.09
Sex		ns		ns		ns
Female	196	3.30 ± 0.03	177	13.6 ± 0.15	177	113.9 ± 1.67
Male	211	3.28 ± 0.03	185	13.9 ± 0.16	185	116.8 ± 1.70
Year		***		***		***
2012	50	3.23 ± 0.08^bcd^	46	14.5 ± 0.43^a^	46	125.3 ± 4.98^a^
2014	60	3.37 ± 0.07^abc^	60	14.5 ± 0.23^a^	60	122.3 ± 2.49^a^
2015	106	3.13 ± 0.04^d^	93	13.9 ± 0.27^ab^	93	117.5 ± 2.83^ab^
2016	119	3.40 ± 0.04^ab^	108	13.1 ± 0.11^bc^	108	108.2 ± 1.26^bc^
2017	41	3.18 ± 0.05^cd^	39	13.2 ± 0.15^bc^	39	111.3 ± 1.59^bc^
2018	31	3.53 ± 0.09^a^	16	12.8 ± 0.50^c^	16	106.3 ± 5.45^c^

BWT, birth weight; WWT, weaning weight; *N*, number of observation.

Means with different superscripts in each subclass within a column differ significantly (*P* < 0.05) from each other.Ns, *P* > 0.05; ***, *P* < 0.001; *, *P* < 0.05.

### Postweaning Growth Performance

The overall least-squares means of six-month (SMWT), yearling live weight (YWT), and postweaning gain of Dorper × Tumele sheep under the smallholder management system was shown in Table 2. The fixed effect like sex, blood level, parity, and season of lambing did not affect the six-month and YWT of lambs. Nevertheless, the year of birth exerted a significant influence on the postweaning growth performance of lambs and lambs born in the year 2012 had higher body weight than the other years.

**Table 2.** Postweaning growth performance of Dorper crosses under extensive management

Sources of variation	SMWT (kg)		ADG2 (g day^−1^)	YWT (kg)		ADG3 (g day^−1^)
	*N*	LSM ± SE	LSM ± SE	N	LSM ± SE	LSM ± SE
Over all	305	17.3 ± 0.09	44.1 ± 1.26	128	23.4 ± 0.16	33.5 ± 1.13
CV(%)	—	8.32	44.8	—	7.76	37.9
Blood level		ns	ns		ns	ns
25%	258	17.3 ± 0.27	44.4 ± 1.39	106	23.3 ± 0.42	32.9 ± 1.29
37.5%	47	17.4 ± 0.34	42.8 ± 2.81	22	23.9 ± 0.58	36.5 ± 2.01
Parity		ns	ns		ns	ns
1	64	17.2 ± 0.32	43.8 ± 2.84	29	24.7 ± 0.54	31.0 ± 2.24
2	98	17.3 ± 0.31	42.9 ± 2.07	45	25.1 ± 0.51	34.4 ± 2.00
3	83	17.5 ± 0.33	43.9 ± 2.70	31	24.9 ± 0.56	33.7 ± 1.99
4	40	17.2 ± 0.38	44.2 ± 3.26	14	24.9 ± 0.68	35.0 ± 3.32
>5	20	17.7 ± 0.41	51.8 ± 3.61	9	24.0 ± 0.70	34.1 ± 6.14
Season		ns	ns		ns	ns
Dry	147	17.6 ± 0.15	46.1 ± 1.91	52	23.7 ± 0.25	34.4 ± 1.99
Main rain	53	17.5 ± 0.18	40.4 ± 2.23	21	22.9 ± 0.35	30.6 ± 1.86
Short rain	105	17.3 ± 0.17	43.4 ± 2.16	55	23.3 ± 0.32	33.8 ± 1.78
Sex		ns	ns		ns	ns
Female	153	17.4 ± 0.30	44.5 ± 1.80	92	24.5 ± 0.45	32.6 ± 1.10
Male	152	17.4 ± 0.29	43.7 ± 1.73	36	24.9 ± 0.52	35.8 ± 2.86
Year		***	***		**	*
2012	20	20.8 ± 0.60^a^	70.2 ± 4.06^a^	—	—	—
2014	58	17.0 ± 0.23^c^	30.7 ± 2.87^d^	33	22.3 ± 0.40^b^	28.4 ± 2.55^b^
2015	83	17.4 ± 0.18^bc^	40.8 ± 2.76^cd^	41	24.3 ± 0.32^a^	37.1 ± 2.12^a^
2016	100	17.2 ± 0.02^c^	45.6 ± 1.42^c^	44	23.5 ± 0.21^ab^	34.7 ± 1.32^ab^
2017	33	17.7 ± 0.11^bc^	51.3 ± 1.73^bc^	10	23.3 ± 0.69^ab^	30.8 ± 4.32^ab^
2018	11	18.2 ± 0.71^b^	57.8 ± 7.91^b^	—	—	—

*N*, number of observation; SMWT, six-month weight; YWT, yearling weight; ADG2, weight gain from 3 to 6 month; ADG3, weight gain from 6 to 12 months of age.

Means with different superscripts in each subclass within a column differ significantly (*P* < 0.05) from each other

Ns, *P* > 0.05; ***, *P* < 0.001; **, *P* < 0.01; *, *P* < 0.05.

### Kleiber Ratios

The KRs of Dorper × Tumele sheep at different ages are presented in Table 3. The overall least-squares means of KR1, KR2, and KR3 were 16.1 ± 0.08, 5.08 ± 0.13, and 3.10 ± 0.09 g/day/kg^0.75^, respectively. The preweaning KRs for crossbred lambs with a 25% exotic blood level were higher than lambs with a 37.5% exotic gene level. However, the postweaning KR estimates were found to be similar for both genotypes. The higher and lower value of the preweaning KR was observed in the third parity and fifth parity, respectively. However, during postweaning age (KR2) lambs born from second-parity and fifth-parity ewes had relatively lower and higher KR, respectively. The year of lambing exerted a significant influence on the preweaning KR (KR1) and postweaning KR (KR2). The lambs born in the year 2012 and 2014 had a higher preweaning KR. The postweaning KR (KR2) for lambs born in 2012 and 2018 was better than in the other years.

**Table 3.** Kleiber ratios (LSM ± SE) for Doper × indigenous sheep

Sources of variation	KR1(g/day/kg^0.75^)		KR2 (g/day/kg^0.75^)		KR3 (g/day/kg^0.75^)	
	*N*	LSM ± SE	*N*	LSM ± SE	*N*	LSM ± SE
Over all	362	16.1 ± 0.08	305	5.08 ± 0.13	128	3.10 ± 0.09
CV(%)		9.52		43.2		33.5
Blood level		*		ns		ns
25%	309	16.2 ± 0.02	258	5.09 ± 0.15	106	3.04 ± 0.10
37.5%	53	15.6 ± 0.26	47	5.01 ± 0.30	22	3.36 ± 0.17
Parity		*		*		ns
1	76	15.9 ± 0.22^ab^	64	5.09 ± 0.31^ab^	29	2.89 ± 0.18
2	113	16.1 ± 0.14^ab^	98	4.95 ± 0.21^b^	45	3.18 ± 0.16
3	101	16.4 ± 0.14^a^	83	4.98 ± 0.28^ab^	31	3.12 ± 0.16
4	46	15.8 ± 0.22^ab^	40	5.14 ± 0.35^ab^	14	3.22 ± 0.26
>5	26	15.7 ± 0.30^b^	20	5.98 ± 0.42^a^	9	3.08 ± 0.49
Season		ns		ns		ns
Dry season	170	16.2 ± 0.12	147	5.27 ± 0.20	52	3.15 ± 0.15
Main rain	69	16.2 ± 0.15	53	4.66 ± 0.24	21	2.91 ± 0.16
Short rain	123	15.8 ± 014	105	5.03 ± 0.23	55	3.12 ± 0.14
Sex		ns		ns		ns
Female	177	16.0 ± 0.12	153	5.15 ± 0.19	92	3.05 ± 0.09
Male	185	16.2 ± 0.11	152	5.02 ± 0.18	36	3.22 ± 0.23
Year		**		***		ns
2012	46	16.6 ± 0.32^a^	20	7.21 ± 0.38^a^	—	—
2014	60	16.4 ± 0.17^a^	58	3.56 ± 0.32^d^	33	2.69 ± 0.22
2015	93	16.3 ± 0.20^ab^	83	4.77 ± 0.31^c^	41	3.34 ± 0.16
2016	108	15.6 ± 0.09^c^	100	5.37 ± 0.16^bc^	44	3.23 ± 0.10
2017	39	16.0 ± 0.10^abc^	33	5.92 ± 0.19^bc^	10	2.85 ± 0.32
2018	16	15.5 ± 0.37^c^	11	6.44 ± 0.70^ab^	—	—

Ns, *P* > 0.05; ***, *P* < 0.001; **, *P* < 0.01; *, *P* < 0.05.

*N*, number of observation; KR1, Kleiber ratio from birth to 3 month; KR2, Kleiber ratio from 3 to 6 month; KR3, Kleiber ratio from 6 to 12 months of age.

Means with different superscripts in each subclass within a column differ significantly (*P* < 0.05) from each other.

### Goodness of Fit of Growth Models

The estimated nonlinear growth model (Gompertz, Logistics, Brody, Monomolecular, and Negative exponential) parameters along with goodness of fit statistics, viz., AIC, BIC, RMSE, and *R*^2^ values of Dorper × indigenous sheep are presented in Table 4. Brody’s model had a lower value of AIC, BIC, and RMSE and a high value of *R*^2^ relative to other growth functions. On the contrary, Monomolecular and logistics models had a high value of AIC, BIC, RMSE, and a low value of coefficient of determination (*R*^2^).

**Table 4.** Estimated growth curve parameters from different models

Model	Growth curve parameters			Goodness of fit			
	*A*	*B*	*K*	BIC	AIC	*R* ^2^	RMSE
Gompertz	21.8 ± 0.13	1.79 ± 0.02	0.41 ± 0.007	1,947	12,255	0.983	1.97
Logistics	20.7 ± 0.11	4.42 ± 0.13	0.66 ± 0.01	2,151	12,459	0.981	2.12
Brody	23.8 ± 0.22	0.85 ± 0.003	0.21 ± 0.005	1,758	12,066	0.985	1.84
Monomolecular	20.7 ± 0.11	1.48 ± 0.03	0.66 ± 0.01	2,151	12,459	0.981	2.12
Negative exponential	22.5 ± 0.21	—	0.28 ± 0.007	2,731	13,044	0.971	2.60

*A*, asymptotic weight; *B*, integration constant or initial animal weight; *K*, maturation rate, BIC, Bayesian information criterion; AIC, Akaike’s information criterion, *R*^2^, coefficient of determination; RMSE, root mean square error.

### Growth curve parameter estimates

**Figure 1.**
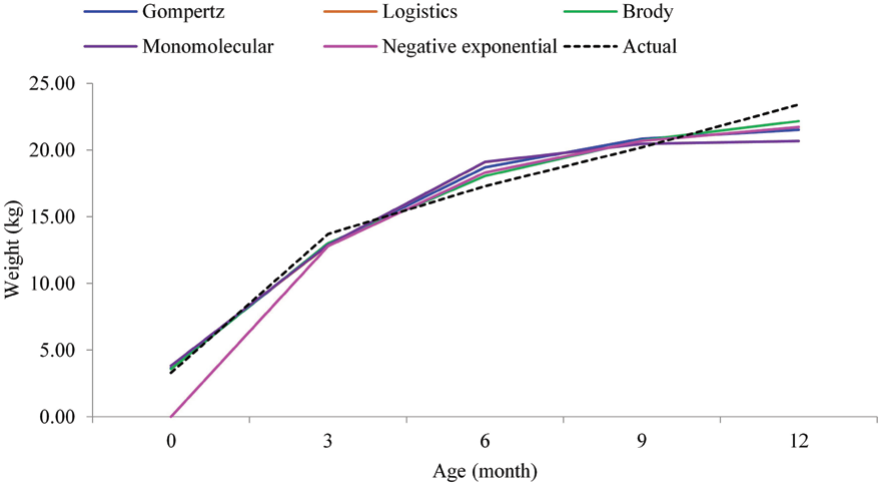
Predicted body weights (kg) as a function of age (months) obtained with different growth models for Dorper x indigenous sheep.

Estimated growth curve parameters from different nonlinear growth functions are presented in Table 4. The highest and lowest value of parameter *A*, which estimates asymptotic weight was observed for the Brody (23.8 ± 0.22) and Logistics (20.7 ± 0.11) models, respectively. Based on the best-fitted model (Brody), the value of parameter *A* was 23.8 ± 0.22 kg. The greatest and lowest value of parameter *B* was observed in Gompert and Brody models, respectively. The value of parameter *K* (maturity rate) ranged between 0.21 and 0.66. The best-fitted model or Brody had a lower value of parameters *B* and *K* relative to other functions. As per the best model (Brody) in this study, the correlation between parameter *A* and parameter *K* was −0.92. The actual and predicted live weight obtained with different growth functions for Dorper × Tumele sheep are shown in Figure 1. The growth rate of crossbred sheep seems excellent to 3 months followed by 6 months of age. However, the growth increased at decreasing rate afterward. Brody model estimated results closer to those actual. However, other models overestimated at 6 months and underestimate at 12 months of age.

## DISCUSSION

### Live Weight and Weight Gain at Different Growth Phases

The BWT of Dorper × indigenous sheep found in the current study is higher than the reports of several scholars (Belete et al., 2015; Abebe et al., 2016; Gemiyo et al., 2017) for Dorper crossbred sheep. Likewise, the WWT of Dorper × Tumele sheep in this study is higher than the reports of several studies (Mekuriaw et al., 2013; Tilahun et al., 2016; Gemiyo et al., 2017; Amare et al., 2018) for indigenous and crossbred sheep. The preweaning gain of Dorper × Tumele sheep observed in this study is lower than the value reported for pure Dorper (142.93 g day^−1^), but higher than the value reported for 50% Dorper × Menz sheep (106.24 g day^−1^) under semi-intensive management (Abebe et al., 2016) and also higher than the result (99.8 ± 2.4 g day^−1^) reported for Dorper × Adilo sheep by Gemiyo et al. (2017). This indicates that the pre-weaning growth performance of Dorper × Tumele sheep was better than other crossbred and non-improved indigenous sheep breeds.

In line with the current result, the nonsignificant influence of sex on preweaning growth traits is noted by Abd-Allah et al. (2012). However, the superiority of males over females was well documented by several reports (Mohammadi et al., 2010; Teklebrhan et al., 2014; Mandal et al., 2015; Abebe et al., 2016). The effect of parity is consistent with Hussain et al. (1996) who noted that weight increases at least up to third parity and according to Mandal et al. (2015), the preweaning growth performance of lambs born from second to third parity dams was higher than lambs of younger and older ewes. The decline of preweaning gain of lambs born from above third parity ewes could be explained by the reduction in milk production performance of their dams. The superiority of lambs born during the main rainy season is consistent with Teklebrhan et al. (2014). The variability of climate conditions and the nutrition available for the dam during the gestation period could be the possible reasons for the observed variation across the season. The considerable influence of the year was noted elsewhere (Berhanu and Aynalem 2009; Teklebrhan et al., 2014; Abebe et al., 2016). The variations across the years may have been due to the variability of rainfall amount and forage abundance.

The SMWT (market weight) of Dorper × Tumele sheep under a smallholder management system was higher than most of the indigenous breeds (Farta, Washera, and Wollo sheep) and even similar to the value reported for 50% Dorper × Menz sheep (17.23 kg) managed under semi-intensive management system (Abebe et al., 2016). The YWT for Dorper × Tumele sheep was comparable with the value reported for Washera and Farta × Washera (Mekuriaw et al., 2013) and Awassi × Tikur sheep (Tilahun et al., 2016). However, the present figure was higher than Farta sheep (Mekuriaw et al., 2013) and Wollo sheep (Amare et al., 2018). This performance difference across studies might be related to the management system and the genetic potential of the breeds.

### Kleiber Ratio

Kleiber ratio shows how an animal grows efficiently and is suggested as an efficient selection criterion for feed efficiency under a low-input production system (Mohammadi et al., 2011), where the feed intake of individual animal is not recorded. The preweaning KR of Dorper × Tumele sheep in this study was comparable with the value reported for Muzaffarnagari sheep (Mandal et al., 2015). However, a relatively lower estimate than the present finding was reported by Mohammadi et al. (2010) for Sanjabi sheep. In agreement with the current finding, Mandal et al. (2015) noted that the lambs born in the second to third parities of the dam had a higher KR than lambs of younger or older ewes at the preweaning stages of development. The superior KR of lambs in the second and third parities indicates that lambs in these parities require less maintenance energy than lambs in the other parities. The higher preweaning and lower postweaning KR of lambs born from early parities could be due to the compensatory growth of lambs during the postweaning age. Sizable influence of birth year on KRs in different development phases was documented by several scholars (Mohammadi et al., 2010; Mandal et al., 2015), which is consistent with the current finding. This variation could be explained by management variation and genetic potential of breeds.

### Growth Curve Parameters

Choosing a poor-fitting model can lead to biologically meaningless growth rates, inflection points, and upper asymptote values (Do and Miar, 2019). Therefore, the choice of an appropriate growth model is essential for understanding animal growth. In this study, five nonlinear growth models were evaluated to identify the most appropriate model. The lower values of AIC, BIC, and RMSE and higher value of coefficient of determination than the other growth functions indicate that Brody was the best-fitted growth function for Dorper × Tumele sheep under a smallholder management system. Similar results were reported in Hemsin sheep (Kopuzlu et al., 2014), Mecheri sheep breed (Balan et al., 2017), Mehraban sheep (Hojjati and Hossein-Zadeh, 2018), Mecheri sheep (Balan et al., 2017), Mehraban sheep (Hojjati and Hossein-Zadeh, 2018), Mengali sheep (Iqbal et al., 2019), and Kordi sheep (Mohammadi et al., 2019). However, this finding disagrees with the report of Ghavi Hossein-Zadeh (2015) who noted the Richards model and Ghavi Hossein-Zadeh (2017) who selected Gompertz function as an adequate function. The variation of selected models among studies could be explained by the management of animals, size or maturity weight, maturity rate of sheep, sample size, and data structure.

Growth curves explain the change in the live weight over time (Owens et al., 1993). Growth curve parameters are immensely important to support the feeding and management decision and for the optimization of sheep production. Hence, due to the economic significance of mature weight, rate of maturation, and related traits, lifetime weight-age associations have attracted the attention of animal scientists and producers (Bilgin et al., 2004; Kopuzlu et al., 2014; Lupi et al., 2016). In addition, the current crossbreeding program aimed to improve growth and meat production, thus, understanding the biology of live weight and age-live weight relationship is of great importance and vital for successful genetic improvement programs. The value of parameter *A* (mature weight) in this study is higher than the value (20.6 ± 2.24) noted for Mecheri sheep (Balan et al., 2017), but lower than the result reported by Hojjati and Hossein-Zadeh (2018) and Malhado et al. (2009). The variation of estimates across studies could be attributed to the difference in breed, due to differences in the size of the skeleton, hormonal status, plane of nutrition (Owens et al., 1993), time unit, and growth models used.

Parameter *K* is the other important parameter that has good biological interpretation and implication. The parameter *K* indicates the earliness of maturing (Lupi et al., 2016) and used to evaluate the relationships between size and productivity of animals. The current estimate for *K* was higher than the report of Kopuzlu et al. (2014) for Hemsin sheep, Balan et al. (2017) for Mecheri sheep, Hojjati, Hossein-Zadeh (2018) for Mehraban sheep, and Mohammadi et al. (2019) for Kordi sheep. Animals with higher *K* values achieved mature weight earlier and animals with lower values achieved mature weight later. Therefore, the current estimate indicates that Dorper × Tumele sheep had a better speed to achieve mature weight than mentioned sheep breeds. Accordingly, it is possible to verify that Dorper × Tumele sheep present a faster growth and can be slaughtered earlier. Besides, rapid growth during the early period can provide more profit to the farmer by reducing the cost of sheep production inputs.

The correlation of parameters *A* and *K* is more important biologically (Hojjati and Hossein-Zadeh, 2018). In addition, these two parameters are important to determine the ideal age of slaughtering with maximum muscle deposition and minimum fat, which could satisfy the requirements of consumers (Amaral et al., 2011). The negative relationship between the two parameters in this study indicates that the early mature crossbred sheep are less likely to exhibit high adult weight. A similar high and negative correlation was observed by Hojjati and Hossein-Zadeh (2018). According to Owens et al. (1993), a larger mature mass is not desired for the breeding herd since animals with higher mature weight need more energy for maintenance and development later in life. The growth curve in this study was Sigmoid type and in line with previous studies (Ghavi Hossein-Zadeh, 2015; Hojjati and Hossein-Zadeh, 2018). In general, due to their relationship to other attributes and the economy of production, growth curve parameter estimates can be crucial for selection and culling of animals.

From this finding, it can be concluded that the growth rate and growth efficiency of crossbred lambs were higher during the preweaning period than the postweaning period. Brody, a model without an inflection point is appropriate to describe the growth curve of Dorper crossbreds under a smallholder management system. The growth curve parameter estimate in this study indicates that Dorper × Tumele sheep had a better speed to achieve mature weight and the early mature crossbred sheep are less likely to exhibit high adult weight. A rapid growth of Dorper crossbreds during early age would improve the income of producers by reducing the cost of inputs. Therefore, crossing Tumele sheep with Dorper sheep integrated with improved management would improve the productivity of sheep and profit from sheep production.

## Data Availability

Data available on request due to privacy/ ethical restrictions.
